# COVID-19 Omicron variant - Time for airborne precautions

**DOI:** 10.1016/j.amsu.2022.103919

**Published:** 2022-06-04

**Authors:** Zohaib Yousaf, Muhammad Arslan Khan, Muhammad Sohaib Asghar, Musharaf Zaman, Mushtaq Ahmed, Muhammad Junaid Tahir

**Affiliations:** a Hamad Medical Corporation, Doha, Qatar; b Department of Pharmaceutical Sciences, University of Lahore Teaching Hospital, Lahore, Pakistan; c Dow University of Health Sciences–Ojha Campus, Karachi, Pakistan; d Lahore General Hospital, Lahore, Pakistan

**Keywords:** Prevention, COVID-19, Variants, Outbreak, Pandemic

## Abstract

Genetic mutations in SARS-CoV-2 have resulted in variants with more transmissibility and partial resistance to COVID-19 vaccines, as seen in the recently classified variant of concern (VOC) “Omicron”. The rapid spread has raised concerns about Omicron being airborne, which leads to a high risk of contamination in public premises, particularly among the frontline healthcare workers. Mandatory usage of protective face masks and respirators is highly recommended in order to break the chain of transmission. Furthermore, health authorities need to reassess the modes of transmission of VOCs and provide updated guidelines to the general public for its prevention.

Coronavirus disease 2019 (COVID-19) is an illness caused by a novel coronavirus now called severe acute respiratory syndrome coronavirus-2 (SARS-CoV-2). SARS-CoV-2 has evolved over time during viral replication due to genetic mutations that have given rise to multiple “variants” all around the world. The center for disease control and prevention (CDC) has defined a variant as “a viral genome (genetic code) that may contain one or more mutations” [1]. According to the World Health Organization (WHO) A variant of concern (VOC) is a variant that results in increased transmissibility or a negative change in COVID-19 epidemiology, or an increase in virulence, or a change in clinical disease presentation, or a decrease in the effectiveness of public health and social measures, or available diagnostics, vaccines, or therapeutics [2]. WHO has recognized five VOCs till-date. A deleterious change in coronavirus disease epidemiology has been determined by the technical advisory group on SARS-CoV-2 virus evolution (TAG-VE). On November 26, 2021, Omicron was designated as a VOC [3].

Recently classified VOC, Omicron, consists of 50 mutations in its viral genome and its spike (S) protein has 30 mutations that brought about transmissibility greater than other VOCs, along with partial resistance to COVID-19 vaccines [4].Consequently, an increased rate of spread was observed, as, in a single week of January 2022, 7 million COVID-19 cases were reported in WHO European Region [5]. This significantly increased rate highlights a concern of Omicron spread via aerosols. Aerosols are breathing particles, ranging in size from 0.5 μm up to 20 μm, that remain suspended in the air. As opposed to aerosols, droplets are breathing particles larger in size and fall to a distance within 2 m. An airborne virus can be rapidly propagated via air if it is present in an aerosol [6]. The rapid transmission of Omicron raises concern for its airborne spread. Considering this factor, varying public premises are at a high risk of contamination that can lead to deleterious consequences if not addressed [7]. Hence, there is a need to reevaluate the modes of VOCs transmission and its prevention in order to combat the arising wave of this infection.

Prevention from inhalation of potentially infectious contaminants has become possible by respirators which are manufactured as personal protective equipment (PPE) [8]. CDC has recommended using of respirators such as N95s and KN95s. Surgical N95s are specific to be used in healthcare settings [9]. Filtering face-piece respirators (FFRs, e.g., N95/FFP2, N99/FFP3) are designed as close-fitting and disposable devices to provide excellent effectiveness against harmful inhaled particles of varying sizes. For example, NIOSH-approved N95 respirators consist of filter media through which at least 95% of particles, with a median count diameter of 0.075 m at a high flow rate of 85 L min–1, can be filtered [10].

In conclusion, SARS-COV-2 cases are potentially rising in multiple countries, resulting due to evasion from immunity from vaccination and higher transmissibility. To cut down the spread of SARS-CoV-2, appropriate PPE is essential requirement for public and healthcare professionals. Health authorities must consistently analyze and update guidelines for the use and selection of PPEs according to the latest findings regarding VOCs. This can help in increasing compliance as well as a reduction in SARS-CoV-2 transmission. Along with the vaccination campaigns, there is a need of raising awareness about appropriate use of PPEs among general public and medical community.

Rate of prevalence and incidence of Covid-19 cases all over the world is given below [11](Fig. 1).

**Fig. 1:**
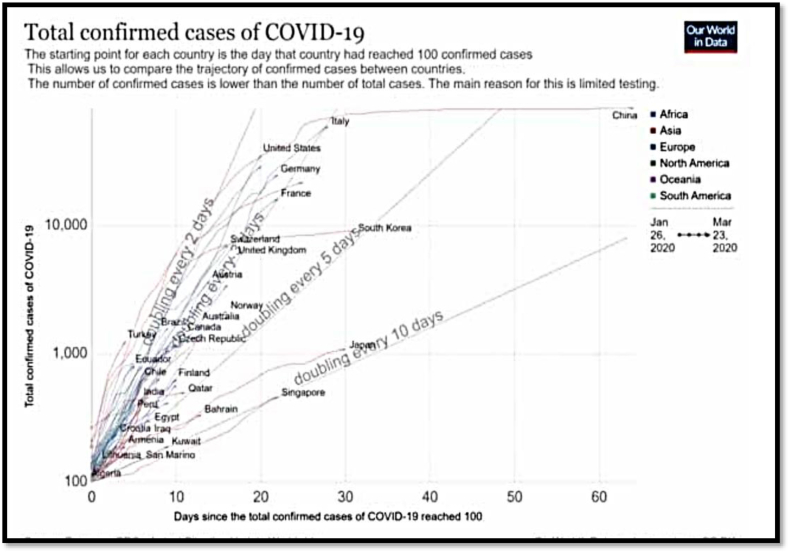
Rate of prevalance and incidence of covid-19.

## Future prospects

To overcome the deadly wave of omicron variant infections, public awareness should be spread regarding its rapid transmissibility. Use of personal protective equipments, circulation of fresh air in indoor settings, and immunization against the pernicious virus should be promoted. Table 1 shows some of the key points for future considerations.

**Table 1:** Future Prospects of omicron variant

Sr. No.	Study	Description
1	Mostafavi E et al. [12]	The newly emerged variant of covid-19, Omicron is considered a variant of concern with high transmission. This study focused on the structure, pathogenesis and management strategies to be opted for controlling this wave of illness.
2	Araf Y et al. [13]	This study discussed the genomics and transmission of omicron as well as effectiveness of vaccine against this new variant of covid-19, omicron.
3	Zheng J et al. [14]	High transmission of omicron variant via aerosols of infected patients
4	Cheng VC-C et al. [15]	Omicron variant is transmitted through airborne route and can be effectively controlled by increased indoor dilution of air.
5	Mohapatra RK et al. [16]	Contaminated air associated with omicron variant with high transmission rate poses a challenge to control its spread.

## Ethical approval

Not required.

## Sources of funding for your research

None.

## Author contribution

Z.Y and M.J.T conceived the idea, M.A.K, Z.Y, M.Z, and M.S.A retrieved the data, did write up of letter and finally Z.Y, M.J.T, and M.S.A reviewed and provided inputs. All authors approved the final version of manuscript.

## Conflicts of interest

None.

## Consent

Not required.

## Registration of research studies

1. Name of the registry: Not required.
2. Unique Identifying number or registration ID: N/A
3. Hyperlink to your specific registration (must be publicly accessible and will be checked):

## Guarantor

Muhammad Sohaib Asghar.

## Provenance and peer review

Externally peer reviewed, not commissioned.

## Financial Support

No financial support was acquired for this article.

## Declaration of competing interest

The authors declare no conflict of interest.
